# Critical care - where have we been and where are we going?

**DOI:** 10.1186/cc11500

**Published:** 2013-03-12

**Authors:** Jean-Louis Vincent

**Affiliations:** 1 Department of Intensive Care, Erasme University Hospital, Université libre de Bruxelles, Route de Lennik 808, 1070 Brussels, Belgium

## Abstract

The first ICUs were established in the late 1950s and the specialty of critical care medicine began to develop. Since those early days, huge improvements have been made in terms of technological advances and understanding of the pathophysiology and pathogenesis of the disease processes that affect critically ill patients. Progress in therapeutics has been less dramatic, but process of care has improved steadily with important changes, including less iatrogenicity, better communication with patients and families, and improved teamwork, which have helped improve outcomes for ICU patients. Critical care medicine is one of the fastest-growing hospital specialties and, looking back, it is clear just how far we have come in such a relatively short period of time. With the ICU set to occupy an increasingly important place in hospitals worldwide, we must learn from the past and wisely embrace new developments in technology, therapeutics, and process, to ensure that the goals of critical care medicine are met in the future.

## 

When I want to understand what is happening today or try to decide what will happen tomorrow,

I look back.

Omar Khayyam

## Introduction

Looking back over the years since the first ICUs were developed, we can clearly see how critical care medicine has developed in terms of technology, with modern respirators replacing the bulky iron lungs of the past, modern ultrasound machines providing instant imaging at the bedside, and modern monitoring systems enabling non-invasive assessment of multiple variables. Particularly striking also have been the improvements in our understanding of diseases and their pathophysiology. Advances in therapeutics have been less dramatic and are less obvious, but are nevertheless present; there have perhaps not been many (or any) dramatic changes that have altered critical care practice overnight, but, rather, evolution has come in a succession of small forward-moving steps [1]. The process of care has also evolved slowly but surely and the changes here have perhaps had the greatest impact on outcomes, with improved teamwork and specialist training, reduced iatrogenicity, earlier patient mobilization, more personal care of the patient and their families, and so forth.

In this article we will look back at where we have come from before briefly reflecting on where critical care medicine is likely to be going in the future.

## Critical care: the past

The concept of critical care and realization of the need for a separate specialty in terms of medical and nursing skills and physical unit position evolved over time as it gradually became apparent that seriously ill or injured patients could benefit from closer attention than was provided to less severely ill patients; this growing realization came at a time when improvements in technology, monitoring, and therapeutics were enabling greater numbers of such patients to survive.

There are several key figures and events commonly associated with the origin of critical care medicine and development of ICUs [2,3], although many other unrecognized individuals have certainly contributed to the development of this field. During the Crimean War in the 1850s, Florence Nightingale demanded that the most seriously ill patients were placed in beds near to the nursing station so that they could be watched more closely, creating an early focus on the importance of a separate geographical area for critically ill patients. In 1923, Dr Walter E Dandy opened a special three-bed unit for the more critically ill postoperative neurosurgical patients at the Johns Hopkins Hospital in Baltimore, MD, USA, using specially trained nurses to help monitor and manage them. In 1930, Dr Martin Kirschner designed and built a combined postoperative recovery/intensive care ward in the surgical unit at the University of Tubingen, Germany. Other surgical units followed these examples, such that by 1960 almost all hospitals had a recovery unit attached to their operating rooms.

During the Second World War, specialized shock units were used to provide efficient resuscitation for the large numbers of severely injured soldiers. In the 1950s, several large polio epidemics, notably in Copenhagen, led to the opening of respiratory units for the many patients requiring mechanical ventilation. In 1958, Dr Max Harry Weil and Dr Hebert Shubin opened a four-bed shock ward in LA County - USC Medical Center, Los Angeles, CA, USA to improve the recognition and treatment of serious complications in critically ill patients. That same year, Dr Peter Safar opened a multidisciplinary ICU at Baltimore City Hospital. Over the next decade or so, ICUs began to be created in hospitals across Europe, the USA, and Australasia. In other countries, ICUs are a more recent development - for example, the first ICU in China was established in 1982 [4].

Early ICUs were somewhat isolated, slightly mysterious, and rather frightening places; staff and visitors (when allowed) were often gowned with protective shoe covers, even masks - adding to the sense of anxiety for the patient and their families. Patients were often heavily sedated to facilitate mechanical ventilation and in the belief that this approach would reduce patient agitation and discomfort. Visiting hours were highly restricted to avoid any increase in physiologic stress for the patient, any interference with the provision of care, and to limit the spread of infection in these vulnerable patients [5].

Many of the initial critical care units were staffed by physicians whose primary specialties were in anesthesiology or internal medicine. Often hospitals had separate surgical and medical ICUs, and some, particularly in the USA, also developed specialty respiratory, cardiac, and neurosurgical ICUs. The majority of units were open, with patients managed by their primary admitting physician, so that different patients on a single ICU would be managed by different physicians. Later, it was realized that many ICU patients had similar problems, regardless of the reason for their critical illness, and that closed units, in which patients were managed by a team of specially qualified intensive care physicians and nurses, provided patients with better care and were associated with improved outcomes [6,7]. The important role of the intensivist in maximizing patient outcomes was also recognized [8], and specialist training programs began to develop as intensive care medicine became a specialty in its own right.

As ICUs began to expand and ever-sicker patients were being admitted and observed, the quantity of critical care research being conducted also increased and understanding of the mechanisms of critical illness progressed rapidly. With continuing developments in technology came more sophisticated life-support and invasive monitoring techniques. Management of the intensive care patient was increasingly interventional. Invasive monitoring systems, notably the pulmonary artery catheter, were widely used. Fluid administration, blood transfusions, oxygen administration, and vasopressors became essential parts of the intensivist's therapeutic armamentarium, although the use of these interventions was often supported by relatively little sound clinical evidence.

## Critical care: the present

The present ICU is unrecognizable from that of 40 years ago in terms of technology. Mechanical ventilators are much smaller, more mobile, and more user-friendly. The development of portable ultrasound units and other noninvasive or less-invasive monitoring techniques has decreased the need for pulmonary artery catheter insertion [9]. The focus of critical care has also shifted somewhat, with patient management becoming less invasive whenever possible, less interventional, and more humane. Despite some initial reluctance, particularly from nursing staff, many units now allow unrestricted or slightly restricted visiting as the benefits of contact with family and loved ones have been recognized [10], although this is not yet a universal finding [11-13]. Nevertheless, units are generally much less strict and more friendly and welcoming for the patient and family than in the past. Improved communication with patients and their families is now part of daily practice and the importance of involving the patient and family in decision-making, especially at the end of life, is also stressed, replacing the more paternal approach of the past [14]. The need for a multidisciplinary approach to patient care is also recognized, and increasingly nutritionists, physiotherapists [15], pharmacists [16], infectious disease consultants [17], and members of other relevant specialties are regularly included in patient rounds. The increasing incidence of microorganisms resistant to currently available antimicrobial agents has led to creation of local, regional, and international surveillance systems to monitor antibiotic resistance and microbiology patterns. Large hospital-wide infection prevention schemes, focusing largely on increased awareness and improved hand-hygiene, have also been established to limit development of nosocomial infections.

The renewed interest in evidence-based medicine in the early 1990s focused a rethink of many accepted practices within the ICU as the lack of solid, high-level evidence for many of these interventions began to be appreciated. Well-designed, randomized trials began to evaluate established procedures, including, for example, the pulmonary artery catheter, blood transfusions, the use of albumin, and so forth. The results from some of these studies suggested that much of the morbidity associated with critical care was, in fact, iatrogenic and that some interventions may do more harm than good. For example, blood transfusion triggers could be reduced to lower levels than the widely used 10 g/dl cutoff value [18]; high tidal volumes were shown to be detrimental [19]; the administration of low-dose dopamine to prevent renal failure was shown to be of no benefit [20]; routine insertion of the pulmonary artery catheter was associated with no benefit and increased complications and costs [21]; and excess sedation was associated with worse outcomes [22]. The development of large national and international critical care consortia - such as the ANZICS clinical trials group, the Acute Respiratory Distress Syndrome Network, and the Canadian Critical Care Trials Group - has facilitated such studies and our evidence base is beginning to expand, with data from large-scale observational studies fueling the continued development of multicenter randomized trials. Importantly, widespread use and availability of Internet technology means that results of such studies are now transmitted more rapidly around the globe.

Whereas trials of established interventions have helped improve patient management, studies of potential new therapies, notably for patients with sepsis, have been frustratingly negative. Repeatedly, new approaches have failed to live up to the promise shown in preclinical or single-center studies: tight glucose control [23,24], moderate-dose steroids in septic shock [25,26], and activated protein C [27,28], to name just a few. Attempts have been made to develop clearer definitions of complex ICU syndromes, such as sepsis [29] and acute respiratory distress syndrome [30], in order to try and reduce heterogeneity in the populations of patients selected for clinical trials and increase the likelihood of demonstrating efficacy. Severity scores [31-33] and staging systems [29,34] have also been proposed to better characterize ICU patients and study populations.

The vast amounts of data generated from the ever-increasing number of studies conducted and published, and a belief that standardizing approaches to patient management may improve patient outcomes, have led to a surge in the numbers of guidelines developed by international groups or societies. Guidelines for sepsis management [35], nutrition [36], red blood cell transfusion [37], ICU design [38], and many other aspects of critical care structure and process have all been published. The use of locally produced or adapted protocols has also been encouraged and these are now present on many ICUs, although the use of checklists, such as FASTHUG [39], may represent a more flexible approach to individual patients, particularly in units with adequate numbers of well-trained staff.

Many hospitals have begun to spread intensive care beyond the fixed walls of the ICU, with the creation of so-called medical emergency teams or rapid response teams. The primary purpose of these teams of intensive care-trained staff is to attend, assess, and provide treatment for deteriorating patients on the ward before they reach a state where ICU admission is needed, thus hopefully improving outcomes and creating more efficient ICU bed usage.

## Critical care: the future

**Figure 1:**
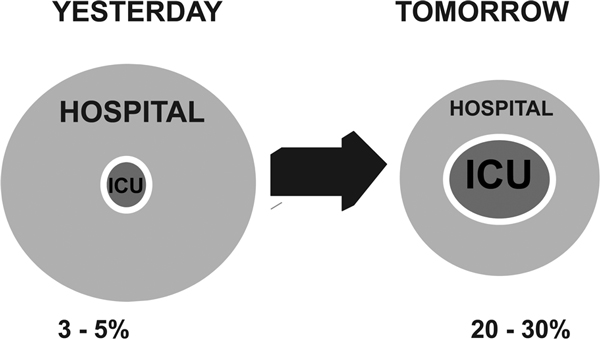
**Changing place of the ICU within the hospital**. Schematic to demonstrate the increasingly large place that the ICU of tomorrow will occupy within the hospital system compared with the past, with ICU beds representing a much larger percentage of total hospital beds.

In 2010 Halpern and Pastores published a review of the evolution in critical care medicine in the United States between 2000 and 2005 [40]. During this time period there was a 4% decrease in the total number of hospital beds, but the number of ICU beds increased by 7%. Hospital non-ICU inpatient days increased by 5%, but ICU inpatient days increased by 10%. Annual critical care medicine costs increased by 44%, but the proportion of hospital costs and national health expenditures allocated to critical care medicine decreased by 1.6% and 1.8%, respectively, over this time period. As the need for intensive care continues to increase, the ratio of ICU beds to hospital beds will continue to rise as the ICU occupies an ever-larger role at the center of acute hospital care (Figure 1).

A key challenge for the future will be to provide adequately trained medical and paramedical staff to cater for the increased numbers of patients, especially as studies have identified large predicted shortfalls in trained intensivists [41]. To deal with the shortages in physician cover, several options have been proposed - including greater use of computerized, nurse-run protocols to manage patients (but the numbers of nursing personnel would then need to be increased to compensate for their extra workload); regionalization of intensive care so that trained staff are concentrated in several larger units, which will provide greater staffing flexibility and may offer improved patient outcomes [42]; and use of telemedicine to enable a trained doctor from a larger institution to provide assistance to less well-staffed, smaller units [43]. A combination of these approaches will probably be needed along with effective admission and discharge criteria to limit use of ICU beds for those who will really benefit from them and financial, academic, and job satisfaction incentives to encourage young physicians to move into critical care [1].

In terms of technology, electronic tools and information technology will play an ever-important role in daily medical practice. Patient records will be immediately available online wherever in the world the patient may find themselves. Prescriptions and tests will be increasingly ordered, viewed, and analyzed via handheld digital assistants or bedside screens, facilitating diagnosis and helping to limit drug errors. Technological advances will clearly play a large part in future medical development, as they have done in the past. Continued developments in genomics, proteomics, and metabolomics will lead to better characterization of patients and their ongoing and underlying disease processes, facilitating diagnosis, prognosis, and therapeutics. These techniques will allow patient care to be increasingly individualized, with treatments chosen according to specific factors within each patient. Continued study will also reveal better treatment targets, which may include microcirculatory measures, cellular markers, and so forth. A multidisciplinary ICU team will become the norm, covering all aspects of patient care from medical therapies and nursing care through to nutritional advice and psychological support. The borders between emergency care and intensive care will become less obvious as early diagnosis and timely resuscitation and treatment move into the pre-ICU arena. Although ICU and hospital survival will of course remain important outcome measures, other more patient-relevant factors, including longer-term outcomes and quality of care measures, will become as important in assessing outcomes. Post-ICU follow-up will also become more common as the negative longer term effects of intensive care are appreciated.

## Conclusion

**Figure 2:**
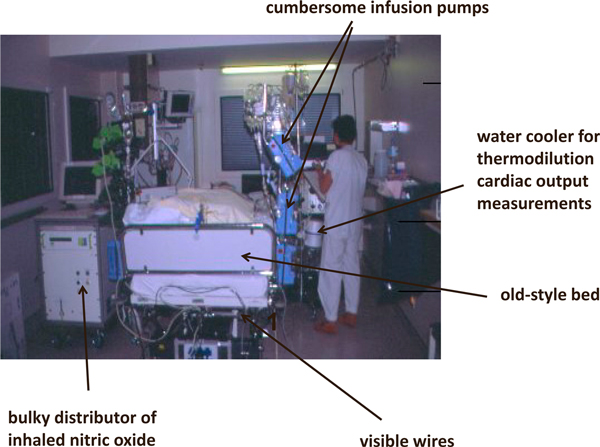
**Our ICU, 20 years ago!**.

Critical care medicine has evolved over the years in terms of structure (Figure 2), process, and outcomes. Fewer and less-invasive interventions, more humane care, earlier diagnosis and treatment, expansion of the service beyond the physical walls of the ICU, and better national and international collaborations with colleagues across the globe are just some of the many changes that we have witnessed since the first ICUs were developed some 60 years ago. Critical care medicine is one of the fastest-growing medical fields in terms of patient numbers, and represents an increasingly important part of healthcare systems in the developed world. Critical care medicine is also more slowly beginning to have a presence in developing countries, and one of the current challenges is to ensure adequate funding, training, and equipment for these newer members of the critical care arena.

We have come a long way but still have some distance to cover as we strive to ensure that critical care medicine provides effective, efficient, evidence-based care to all who need it.

## Competing interests

The author declares that they have no competing interests.

## Declarations

This article has been published as part of *Critical Care *Volume 17 Suppl 1, 2013: Future of Critical Care Medicine. The supplement was proposed by Fresenius Kabi based on presentations from the 'Future of critical care medicine (FCCM) 2012: Today's practice and a look to the future' symposium. Articles were commissioned by the journal, were independently prepared by the authors and have been peer reviewed by the journal. The supplement was edited by John Marini, who declares that he has no competing interests. Publication of the supplement was supported by Fresenius Kabi.
